# A perspective on the molecular simulation of DNA from structural and functional aspects

**DOI:** 10.1039/d0sc05329e

**Published:** 2021-03-15

**Authors:** Manas Mondal, Lijiang Yang, Zhicheng Cai, Piya Patra, Yi Qin Gao

**Affiliations:** Institute of Systems and Physical Biology, Shenzhen Bay Laboratory 518055 Shenzhen China gaoyq@pku.edu.cn; Beijing National Laboratory for Molecular Sciences, College of Chemistry and Molecular Engineering, Peking University 100871 Beijing China; Biomedical Pioneering Innovation Center, Peking University 100871 Beijing China; Beijing Advanced Innovation Center for Genomics, Peking University 100871 Beijing China

## Abstract

As genetic material, DNA not only carries genetic information by sequence, but also affects biological functions ranging from base modification to replication, transcription and gene regulation through its structural and dynamic properties and variations. The motion and structural properties of DNA involved in related biological processes are also multi-scale, ranging from single base flipping to local DNA deformation, TF binding, G-quadruplex and i-motif formation, TAD establishment, compartmentalization and even chromosome territory formation, just to name a few. The sequence-dependent physical properties of DNA play vital role in all these events, and thus it is interesting to examine how simple sequence information affects DNA and the formation of the chromatin structure in these different hierarchical orders. Accordingly, molecular simulations can provide atomistic details of interactions and conformational dynamics involved in different biological processes of DNA, including those inaccessible by current experimental methods. In this perspective, which is mainly based on our recent studies, we provide a brief overview of the atomistic simulations on how the hierarchical structure and dynamics of DNA can be influenced by its sequences, base modifications, environmental factors and protein binding in the context of the protein–DNA interactions, gene regulation and structural organization of chromatin. We try to connect the DNA sequence, the hierarchical structures of DNA and gene regulation.

## Abstract 2

An overview of atomistic simulations on the effect of sequences, base modifications, environmental factors and protein binding on DNA’s hierarchical structure and dynamics in the context of protein–DNA interactions, gene regulation and structural organization of chromatin.

## Graphical abstract

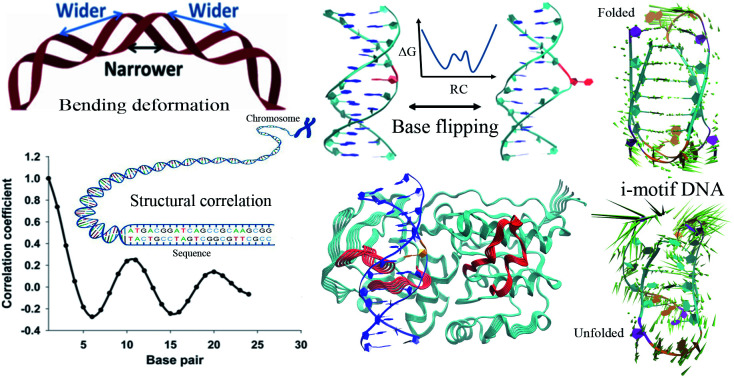

## 1. Introduction

As genetic material, DNA carries structural information in its primary sequence, which controls the correct duplication and regulates the expression of hereditary information. Biological processes ranging from replication and transcription to gene regulation, base modification and DNA repair are associated with the sequence-specific structure, deformability and dynamics of DNA. To initiate and regulate these different biological processes, the recognition mechanism and sequence-specific interactions of DNA with proteins or small molecules are prerequisite.^1–4^ In cells, gene regulatory networks have emerged as central elements of regulation and the recognition specificity of DNA sequences is the key for understanding cellular functions. The base sequence of polynucleotides significantly affects the characteristic three-dimensional structure and flexibility of DNA.^5–9^ Furthermore, the sequence-dependent flexibility and dynamics of DNA are also governed by its three-dimensional structure, which is relatively complex and maintained by different covalent and non-covalent interactions between its building blocks and surrounding environment.^10–12^ Environmental factors such as solvent, temperature, pH and salt concentration can influence the structure and dynamics of DNA in subtle ways. Under physiological conditions, DNA can adopt a wide range of conformations, although the predominant structural form of DNA is the antiparallel right-handed double helix, as described by Watson and Crick.^13^ Due to its polymorphic nature, the double helices of DNA can have several forms depending on the environment, among which A-DNA and B-DNA are common. In addition to the canonical double helical structure maintained by Watson–Crick base pairing, numerous stable non-canonical configurations have been identified, which are stabilized by various hydrogen bonding patterns besides Watson–Crick pairing.^12,14–17^ For example, the telomeric G/C-rich regions of eukaryotic chromosomes, which contain highly conserved short tandem repetitive DNA sequences, can form the G-quadruplex (G4)/i-motif structure.^12,18–20^ G-quadruplex/i-motif-forming sequences are also found in gene promoter regions of several oncogenes, which can control gene regulation, induce genetic instability, and consequently, cause human diseases.^19–22^ Besides the structure of DNA, the packaging of DNA in cells is also important in the realization of all types of biological functions. For example, the compact forms of long DNA, such as supercoils, knots, chromatin and chromosomes, are central to fundamental mechanisms for replication, transcription and recombination.^23–25^

The importance of the physical properties of DNA has long been implicated in chromosome organization, from nucleosome positioning to DNA–DNA interaction influencing the structure of chromatin at the genome scale.^26–28^ DNA is a highly charged polyelectrolyte, where in ionic solution, an approximately 40–250 Mb long sequence of one human chromosome will spontaneously adopt a random coil conformation. Inside the cell nucleus, 46 of these chromosomal polynucleotides are compacted to fit a volume of approximately 10 μm in diameter. During compaction, how this polyelectrolyte can overcome the entropy loss and resist repulsion between the like-charged chain segments to fit inside the small volume of the cell nucleus is not clearly understood. In an elementary building block of eukaryotic chromatin, the nucleosome, only half of the DNA charge is neutralized by the positive charges on histone proteins. However, little is known about the composition of species neutralizing the rest of the DNA charges. Obviously, Mg^2+^, K^+^, Ca^2+^, and Na^+^ should be the primary cations that can neutralize the charges of the DNA phosphate backbone.^29–32^ The sequence-dependent structure, flexibility, and biological function of DNA are heavily influenced by the large cloud of negative potential generated around its double helix by the charged backbone phosphate groups. The major condition for DNA compaction and chromatin folding is sufficient neutralization of the negative charges of DNA by oligo- or mono-valent cations and protein binding.

The conformational dynamics and thermally induced fluctuations of DNA in the local helical conformation, such as bending and base pair opening, which occur on timescales ranging from picoseconds to milliseconds, play a significant role in the biological functioning of DNA. The dynamics of DNA affect specific protein–DNA binding by enabling proteins to indirectly probe the base sequence *via* local changes in mechanical and dynamic behavior. Numerous DNA repair proteins have been found to perform specific base repair extra-helically with the lesion base flipped out of the DNA duplex.^33–35^ The dynamics of this outward base flipping, which is referred to as ‘enzymatic base flipping’, is responsible for the search of damage in the target DNA base by repair proteins, and therefore is fundamental and of great interest. However, the biophysical nature of enzymatic base flipping is still under debate. In one damage search mechanism, it is proposed that the protein binds and then slides through the duplex DNA, and physically tests each base pair in the intra-helical conformation until the unstable lesion base or non-Watson–Crick base pair is recognized.^36,37^ Alternatively, another mechanism postulates that owing to the weakened intra-helical base pairing, the damaged or mismatched base spontaneously flips out of the DNA duplex with a certain probability, and consequently the protein recognizes and captures the fully flipped-out base in the extra-helical conformation for further chemical processing.^38–40^ In both cases, weakened base pairing allows the repair proteins to effectively locate the lesion base, but whether the lesion/mismatched base is recognized in the intra-helical or extra-helical conformation strongly depends on how fast the lesion/mismatched base pair can spontaneously flip out and how long it can stay outside in the absence of DNA repair proteins.^37,38^ The concept of dynamic enzyme–DNA recognition has been developed based on the experimentally determined structures of many enzyme–DNA complexes, which contain highly distorted DNA and sometimes protein such as DNA polymerases, nucleotide excision repair factor UvrA2B, transcription factors, restriction endonucleases, DNA glycosylases, AP endonucleases, DNA methyltransferases, and DNA demethylases.^1,2,41,42^ However, it is still difficult for experimental studies to provide the atomistic details of the interactions, conformational dynamics and changes involved in the different biological processes of DNA.

Recent developments in computational power, advanced simulation approaches and improvements in molecular mechanics force fields have opened up a new horizon in the computer simulation of biomolecular systems to explore the microscopic basis of the structure, dynamics, interactions and functions of biomolecules in different lengths and timescales. Computer simulations are becoming highly complementary to experimental tools, and methods such as all-atom molecular dynamics simulation can provide atomistic descriptions that are typically inaccessible to experiments.^3,4,8,31,43,44^ In this perspective, we provide a brief overview of the atomistic simulation studies on how the hierarchical structure and dynamics of DNA are influenced by its sequence, base modifications, certain environmental factors and protein binding in context of protein–DNA interactions, gene regulation and structural organization of chromatin. There are numerous reports on simulations related to DNA and chromosomes, but due to the limit of the scope of this perspective, we focus on a few topics that have been presented in our recent simulation studies. We first discuss the atomistic studies of the DNA base pair opening or base flipping mechanism and recognition of modified DNA bases in the active site of polymerase. Subsequently, we describe the efforts towards an understanding of the relation between the local/global DNA structural changes and mechanism of DNA allostery. We further discuss the conformational dynamics and gene regulatory function of non-canonical DNA with particular focus on the structural and functional aspects of i-motif DNA, and finally we present an outlook on the molecular basis of the structural organization of chromatin. In general, we try to connect the DNA sequence, the hierarchical structures of DNA and gene regulation.

## 2. The mechanism of DNA base flipping and modification of DNA bases

Deformation of double helical DNA is expected to affect its interactions with proteins. Amongst the deformations of the double helix, base pair disruption is necessary for fundamental biological processes such as DNA replication, transcription, modification and repair.^33,45,46^ Disruption of the hydrogen bonding within a base pair is the first critical step of base flipping, where either base moves away from the DNA double helix. Studies have shown that flipping of the target base from the intra-helical to extra-helical position is a common strategy for a variety of DNA-modifying enzymes, such as methyltransferases, glycosylases, and endonuclease.^33,45,46^ Several crystal and NMR structures^41^ have been reported with flipped DNA base inside the active site of the enzyme; however, these structures cannot provide deep insight into the conformational changes occurring during the flipping process. In the presence of an enzyme, protein–DNA interactions may play important roles in initiating the base flipping process.^33,45–47^ Alternatively, spontaneous base pair opening may be the trigger for further enzymatic interactions.^38,39^ However, in each case, characterizing the base flipping process in terms of DNA only is necessary for fully understanding the more complex scenario of DNA in an enzyme environment. Thus, the exact mechanism for base flipping and the possible transient role of the enzyme in this process is still not well understood at an atomistic level. Although experimental studies cannot provide microscopic details about the energetic preference, enzymatic activity and conformational changes involved in the flipping process, advanced computational simulation studies can provide more insight into these aspects. Therefore, there has been substantial amount of computational effort in this direction.^39,43,47–49^

Spontaneous base flipping is found to be a relatively rare event and difficult to probe. Nevertheless, many efforts have been made experimentally to monitor this process. For example, Spies and Schowen applied an extra-helical base trapping strategy to obtain the spontaneous base flipping rate^50^ and efforts have also been made to measure the imino proton (in each G or T base) exchange rate using NMR spectroscopy.^51,52^ According to these NMR studies, the lifetime of the extra-helical state is in the order of microseconds, and that of the intra-helical state ranges from milliseconds to hundreds of milliseconds, depending on the stability of the individual base pairs. It was found from the base pair opening-closing kinetics that the inner pairs of nucleic acid structures open one at a time. In case of B-DNA, its lifetime ranges from 0.5 to 7 ms and 5 to 50 ms at 15 °C for the d(A.T) and d(G.C) pairs, respectively, and its dissociation constants from 10^−5^ to 10^−7^.^53,54^ The base pair kinetics was also found to depend on the oligonucleotide structure, as characterized in the case of B-DNA, Z-DNA, triple helixes, RNA and i-motif structures.^53^ According to a previous NMR study, the lifetime of the r(G.C) pairs is ∼ 40 to 50 ms, which is longer than that of their equivalent in the corresponding oligodeoxynucleotides, and the dissociation constants of about 10^−7^ are slightly smaller.^54^ It was also found that the r(A.U) pair opening and closing rates are much larger than that of the d(A.T) pairs, but their stabilities are comparable.^54^ NMR studies also showed that the structural variability and dynamics of RNA are mostly associated with the base pair kinetics and stability of different types of base pairing patterns besides canonical Watson–Crick base pairing.^55^ In 2014, Yin *et al.* implemented the PET-ddFCS assay to investigate the dynamics of spontaneous single-base flipping in a mismatched base pair in a DNA duplex.^39^ A single-exponential decay with the characteristic time of ∼10 ms was clearly seen in the ddFCS curve of the G–T mismatch containing DNA, which is associated with the spontaneous flipping of G in the mismatched pair. Similarly, single-exponential decays with the relaxation time of ∼20 ms were found in the correlation functions of both the T–T and C–T mismatches.^39^ However, the probability of the spontaneous outward flipping was found to be small for all the tested mismatches. The bases showed preference to stay inside the double helix even for mismatched pairs. The inward base-flipping rates (the lifetime of the extra-helical base) of all three mismatches were found to be the same, whereas the outward flipping rate (the lifetime of the intra-helical base) of the G–T mismatch was found to be notably slower than that of the T–T and C–T mismatches because of the lower activation energy of the extra-helical state of G–T mismatch.^39^ It was found that the equilibrium constant of the mismatched bases to spontaneously flip out of the DNA duplex increased as the temperature increased, and both the out and inward flipping of the G–T, T–T, and C–T mismatches also sped up as the temperature increased.^39^ Computer simulations also plays an important role in investigating the base flipping mechanism in this context.^39,49^ In evaluating NMR measurements through computer simulations, MacKerell and coworkers and others found that the target imino proton on the base becomes accessible to the solvent for proton exchange when the base pair opens to an angle of only 30°, which is still within the potential well of the hydrogen bond.^49,56^ Free energy simulation studies provide plenty thermodynamic information about base flipping. For instance, Ma *et al.* studied the energetic coupling between DNA bending and flipping of a central thymine in double-stranded DNA 13mers.^57^ Free energy simulations were also used to quantify the equilibrium free energy difference between the closed and open states, energetic difference of flipping toward the major or minor groove side and the free energy barriers for flipping. Base flipping was found to involve much higher activation energies than simple helical deformations, typically in the order of 10–20 kcal mol^−1^, and consequently occurs at much longer timescales (in the order of tens of milliseconds).^33,43,47,49,57^ Because of the high free energy barriers involved in the base flipping process, enhanced simulation algorithms are required for efficient sampling and to help access the structural changes in complex biological processes with long time scales.^48,49,58–60^ The method of choice is typically molecular dynamics (MD) combined with umbrella sampling (US) or other enhanced sampling approaches. Furthermore, numerous strategies have been used to understand the base flipping mechanism in the case of DNA modification, mismatch and damage recognition by DNA glycosylases and other DNA repair enzymes.^39,48,49,61,62^

US simulations are among the most widely used methods in studies on the sequence-dependent DNA base flipping mechanism, in particular the flipping of cytosine (C) in the case of DNA methylation by DNA methyltransferases.^43,47,49^ Lu Jin *et al.* studied the conformational transition of HhaI methyltransferase and flipping of target C into the enzyme active site using metadynamics simulations and proposed an “induced-fit” model to illustrate the base flipping process in M.HhaI.^48^ The time-independent nudged elastic band (NEB) method and US simulation strategy together with Hamiltonian replica exchange simulations were used to study the base flipping process and discrimination mechanism between the oxidative DNA lesion, 8-oxoguanine (oxoG) and its normal counterpart guanine by the repair enzyme DNA glycosylase.^62^ The free energy profile and energetic preference for base flipping show that the enzyme can discriminate against G in favor of oxoG in the early stages of base flipping, and the enzyme can recognize 8-oxoG through multiple gating intermediates in the base eversion pathway. US with Hamiltonian replica exchange simulations were also employed to elucidate the recognition mechanism of the photo-induced *cis-syn*-cyclobutane pyrimidine dimer (CPD) lesion between adjacent thymines in DNA.^61^ The simulations indicated that in the unbound state the free energy penalty for flipping a CPD lesion from the intra-helical state to an extra-helical conformation is lower compared to regular undamaged DNA. However, the calculated free energy penalty of 8 kcal mol^−1^ is still too high to allow frequent spontaneous flipping.

**Fig. 1:**
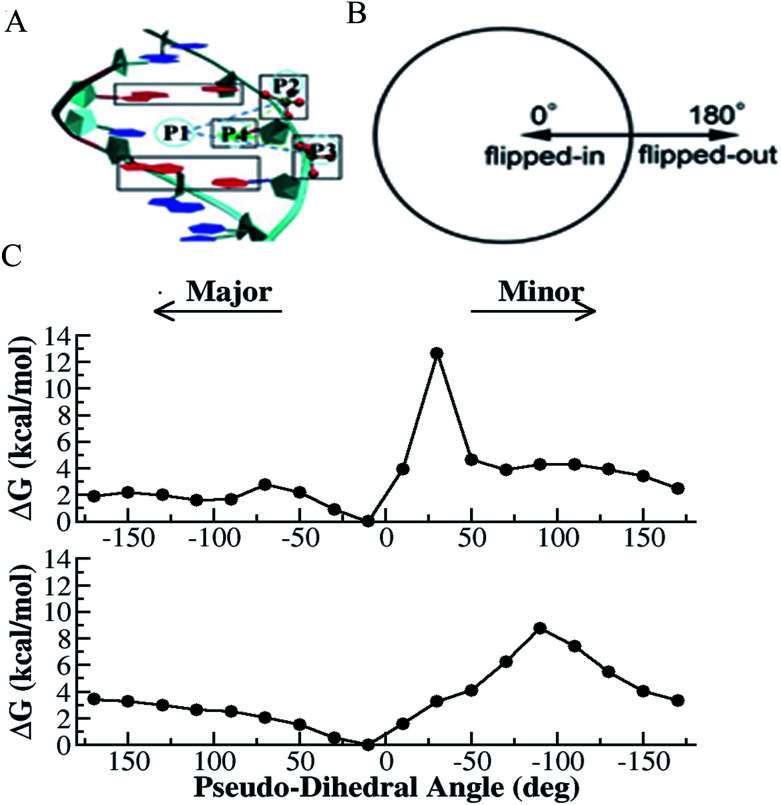
Selective integrated tempering sampling of the mismatched T–T bases (thymine on the right strand named as T1 and the one on the complementary strand named as T2). (A) Definition of the pseudo-dihedral for T1 base flipping. P1 is the center of mass of the two flanking base pairs (red) above and below the flipping T base, P2 and P3 are the center of mass of the phosphates flanking the flipping base, and P4 is the center of mass of the five-member ring of the purine. The dihedral angle is made of the two triangular planes, which share a common side defined by P2 and P3. The base opening angle is defined by the dihedral angle. A similar definition was made for T2 base flipping. (B) Schematic illustration of the correspondence between the defined pseudo-dihedral in A and the states (flipped-in and flipped-out) of a mismatched base. (C) Calculated PMF for T1 (top panel) and T2 (bottom panel) flipping.

To gain detailed atomistic insight into the dynamics of the spontaneous flipping of a single mismatched base pair, we performed selective integrated tempering sampling (SITS) molecular simulations on a dsDNA molecule containing a single mismatched T–T/G–T base pair.^39^ SITS^59,63–65^ can enhance the sampling in the energy and configuration space of the system without any previous information of the reaction coordinates. In the SITS simulations, the system was divided into two sub-regions, the central group, which contains only the flipping T–T/G–T base, and the bath, which includes all the other bases, electrolytes, and water molecules. The system temperature was maintained at 300 K and 100*β* values evenly distributed from 280 K to 600 K were selected to generate the effective potential (eqn (1)) for the enhanced sampling of the flipping of the T/G base:^39^1
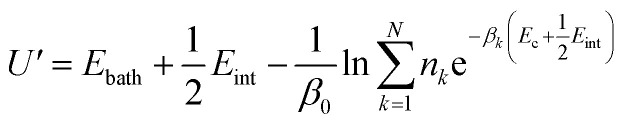
where *E*_c_, *E*_bath_, and *E*_int_ are the potential energies of the central group, the bath, and the interactions between the central group and the bath, respectively. The generated effective potential can represent the approximate behavior of the model system, and for quantitative measures and qualitative information, multiple simulations needed to be performed with considerable caution. A total of 800 ns enhanced sampling simulation trajectories were collected. We employed a previously used pseudo-dihedral angle (CPDb)^66^ as the reaction coordinate and could obtain the free-energy profiles of flipping for both the G–T and T–T mismatches.^39^ The results calculated for T–T mismatch are shown in Fig. 1. We found that the flipping of one base is much more likely than simultaneous flipping of both bases, which can be expected from the stacking energy costs. In Fig. 1(A), the global minima correspond to the T base-embedded states (−10° and 10° for T1 and T2, respectively). Compared to the normal Watson–Crick base pairs, the abnormally paired bases (*i.e.*, T1 and T2) have relatively low free-energy barriers (∼2–3 kcal mol^−1^) along the transition from the intra-helical to the flipped-out states. These computational results confirmed the higher probability of base flipping for the mismatched base pair in the DNA duplex compared to normal Watson–Crick base pairs.^47^ In addition, the spontaneous flipping of either T1 or T2 was shown to occur mainly through the major groove because of the much lower free-energy barrier along this pathway (Fig. 1(C)). Another interesting observation was that the potential of mean force (PMF) profiles of the two thymines, T1 and T2, were distinctly different. Given that the two bases are both thymine, their difference is likely caused by different local environments (*e.g.*, the different adjacent bases for T1 and T2). The different behavior of the two thymines can be used to explain the non-single-exponential relaxation observed experimentally.^39^

**Fig. 2:**
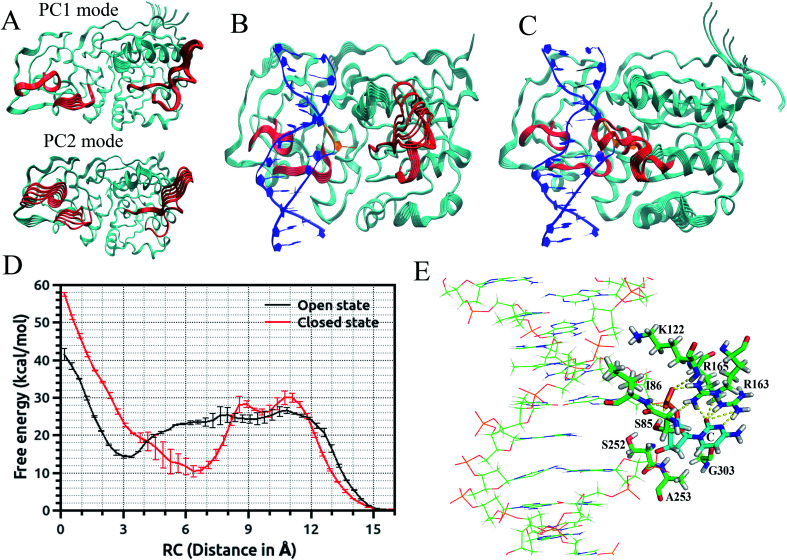
(A) Conformational fluctuation of M.HhaI along the first two-principle component mode. Trajectory of protein motions in the DNA–M.HhaI complex along the first principle component mode: (B) in the open complex and (C) in the closed complex. Target recognition loops and catalytic loop are colored red and average DNA structure and flipped base are shown in blue and orange, respectively. (D) Major groove free-energy profiles for eversion of target C in the DNA–M.HhaI complex in the closed and open state of the protein. Error bars reflect the differences between two independent runs. (E) Active site conformation of target C in the DNA–M.HhaI closed complex with an eversion distance of 15 Å.

Recently, we employed an enhanced sampling strategy by combining the SITS method^59^ with US simulation to study the energetics and base flipping pathway of target C in DNA methylation by DNA methyltransferases (MTases).^67^ The crystal structures of bacterial cytosine 5-methyltransferase M. HhaI complexed with different DNA molecules indicate that the positioning of the target cytosine base in an extra-helical position is essential for chemical modification and catalytic reaction.^41,45^ The formation of the ternary M. HhaI–DNA–AdoMet complex is a subsequent discrete step in the physiological methylation reaction. A previous NMR study suggested that M. HhaI does not accelerate base pair opening beyond the intrinsic rate of DNA breathing, which corresponds to a lifetime for G–C base pairs of ∼10 ms.^53^ The interaction of DNA with the enzyme in the binary complex led to a dynamic equilibrium between an ensemble of flipped-out states of the target nucleotide and the stacked state. However, although several experimental and theoretical simulation studies have been conducted, the base flipping pathway, with the active or passive involvement of the MTase in base flipping and the protein elements that mediate base flipping, are not well understood. In the US simulations, we employed the recently proposed eversion distance^62^ as the reaction coordinate for flipping of target C. The starting flipped conformation of target C is defined by an eversion distance of ∼16.0 Å (as found in the crystal structure of the M. HhaI–DNA complex). The US windows were evenly spaced along the eversion distance at intervals of 0.4 Å, restrained by a 10 kcal mol^−1^ Å^−2^ umbrella potential from 0 to 16 Å, yielding a total of 41 windows in the free energy profile along the major or minor groove. It was found that the flipping of the target base into the active site of the enzyme involves the recognition of cognate sequence 5′-GCGC-3′ through target recognition loops and extensive protein open to closed catalytic conformational changes, resulting from the closure of the conserved catalytic loop. We found that MTase undergoes breathing motions in its free state due to the conformational fluctuation of the catalytic loop and target recognition loops (Fig. 2(A)).^67^ To capture the motions of the catalytic loop and target recognition loops in the base flipping pathway, sampling over the configuration space of selective loop regions of the enzyme in the M.HhaI–DNA complex was achieved using SITS without perturbing the rest of the system. In the SITS simulations, the system temperature was maintained at 300 K and 40*β* values exponentially distributed from 280 to 350 K were selected to generate the effective potential to enhance the sampling of the selected loop configuration.

The US + SITS approach allowed efficient sampling of the target base in high energy regions of the flipping free energy surface, which is difficult to sample in the standard US.^67^ Spontaneous base flipping in DNA in aqueous solution was found to involve large energetic penalties of ∼18 kcal mol^−1^ or more. A previous US study showed that flipping of target C in the active site of M. HhaI is favourable along the major groove in the closed conformation and M. HhaI lowers the free-energy barrier to flipping by ∼17 kcal mol^−1^ and stabilizes the fully flipped state.^47^ We showed that the conformational properties of DNA and correct positioning of the key loop residues in the DNA-protein complex have a strong influence on the loop motions and overall conformational fluctuations of the protein (Fig. 2(B) and (C)). The free energy profiles for the flipping of target C into the enzyme active site supports the major groove base eversion pathway as the dominant path. The results also showed that the closed state of the enzyme increases the free energy barrier, whereas the open state reduces it (Fig. 2(D)).^67^ The closed M. HhaI-DNA complex favors the extra-helical everted state by about 22 kcal mol^−1^ with respect to the intra-helical state and the open complex favors the complete everted state by about 14 kcal mol^−1^ over the intra-helical state at an eversion distance of 3 Å. However, the flipped-out base in the open complex is about 8 kcal mol^−1^ less stable than that in the closed complex.^67^ Thus, the stability of the flipped-out base in the active site of the enzyme is associated with the closing of the catalytic loop. Previous experimental studies based on fluorescence and NMR spectroscopy revealed that the closing of the catalytic loop depends on the recognition of correct binding sites and the open to closed transition does not occur with noncognate DNA.^68^ In our study, the free energy profile for the flipping of mC in the open complex indicates that everted mC can be entrapped in the intermediate state of the base eversion pathway and the steric clash of the methyl group of 5-methyl cytosine with the protein most likely disrupts the formation of the closed catalytic complex.^67^ Therefore, flipping of target base into the enzyme active site pocket and alteration of protein fluctuations assist in forming the closed catalytic complex by stabilizing the flipped conformation of the target cytosine in the catalytic site (Fig. 2(E)). Recent studies showed that the modulation of the protein dynamics and conformational entropy can play an important role in functional ligand binding, protein–protein and protein–DNA interactions associated with complex biological processes.^69,70^ In this context, our study also elucidated the roles of the conformational fluctuations of protein in the base flipping mechanism and catalytic complex formation. Our results support an induced-fit and site-specific DNA recognition mechanism,^48^ where the enzyme searches along DNA for its specific recognition site predominantly in a loop open position and the catalytic loop closes very rarely during the search process or only closes when the target sequence is found.

**Fig. 3:**
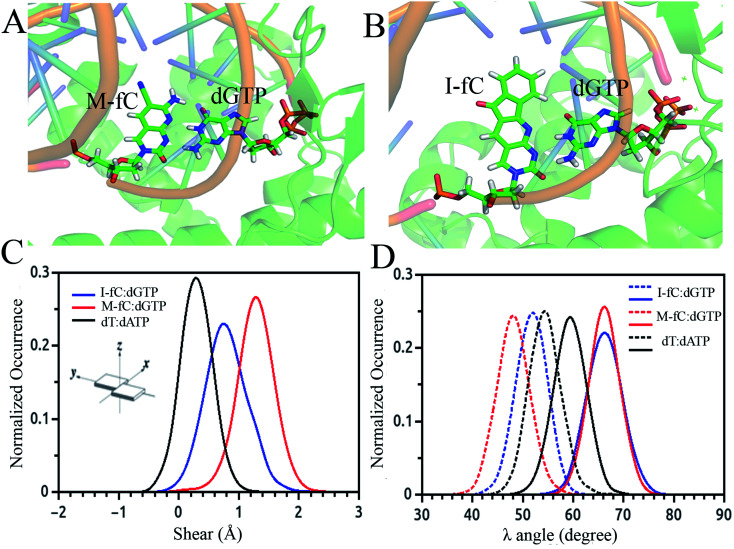
Simulated average structure of the (A) M-fC:dGTP and (B) I-fC:dGTP pairs in the incorporation site of the ternary DNA polymerase. Distribution of parameters for the base pair between the incoming nucleotide and template M-fC/I-fC as obtained from the simulated trajectories: (C) shear and (D) *λ* angle.

Besides the epigenetically modified base 5mC studied above, a variety of other naturally occurring DNA base modifications to the canonical bases serve as epigenetic markers as they have the potential to affect the structure, recognition and function of DNA. Generally, it is difficult to distinguish these modified bases from the original canonical ones by conventional sequencing as most of the modifications, such as 5-methylcytosine (5mC) and N6-methyladenine (6 mA), do not change the Watson–Crick base pairing. Then an interesting question was raised: how are the modified nucleotides recognized by DNA polymerases? In this respect, many unnatural base pairs have been designed and synthesized to clarify the nucleotide recognition mechanism of DNA polymerases from various aspects. Recently, we investigated two unnatural DNA bases, namely M-fC (malononitrile-modified 5-formylcytosine) and I-fC (1,3-indanedione-modified 5-formylcytosine), to understand the mechanisms by which DNA polymerases faithfully decode chemical information on the template. The two unnatural bases are derived from 5-formylcytosine (5fC), which is one of the oxidized derivatives of 5mC and is involved in active DNA demethylation. It was found that the two organic molecules, malononitrile and 1,3-indanedione, could react with 5fC specifically and produce the two unnatural DNA bases M-fC and I-fC, respectively. These two modified cytosine analogs are read as T instead of C during PCR and lead to an almost total C-to-T transition. Based on these phenomena, two 5fC sequencing methods have been developed for bulk samples and at the single cell level.^71^ However, it is difficult to reconcile the C-to-T transition with the accepted mechanism of DNA polymerase recognition. To understand the molecular basis of the incorporation of nucleotide deoxyadenosine triphosphate, dATP, corresponding to modified cytosine (M-fC/I-fC) in the template strand, crystallographic studies were performed together with MD simulations of a ternary complex of polymerase bound to DNA duplex with the respective modified cytosine (M-fC/I-fC) in the template strand and dATP in position poised for catalysis, leading to their incorporation.^72^ The template M-fC/I-fC can form a Watson–Crick-like geometry with the incoming dATP, similar to the geometry observed in the cognate dT:dATP base pair with a similar C1′–C1′ inter-nucleotide distance and *λ* angles. Thus, the presence of the unnatural cytosine in the template strand is capable of inducing the formation of a Watson–Crick-like pair with an incoming dATP at the incorporation site of the DNA polymerase.^72^ Considering that M-fC and I-fC specifically pair with dATP, but not dGTP (deoxyguanosine triphosphate), we then investigated the discrimination mechanism against dGTP. It became apparent from our computational analysis that dGTP shears significantly along the major groove (1.5 Å) and forms a stable wobble base pair with M-fC/I-fC (Fig. 3(A)–(C)). An important feature that distinguishes Watson–Crick pairs from wobble pairs is the symmetry of the *λ* angle.^73^ In the case of the M-fC/I-fC:dATP pair, the *λ* angles are symmetrically distributed, resulting in symmetric *λ* angles of a cognate dT:dATP pair.^72^ However, in the case of the sheared M-fC/I-fC:dGTP pair, the *λ* angle distribution becomes asymmetric (Fig. 3(D)). We proposed that the formation of Watson–Crick pairs at the active site of DNA polymerase is a prerequisite for the incorporation of incoming nucleotides given that dGTP cannot form a cognate base pair with the unnatural cytosines in the active site, and hence may be regarded as a mismatched nucleotide for incorporation by DNA polymerases. Preferential incorporation of dA by DNA polymerases corresponding to a modified or lesioned base in the template strand has been referred to as the A-rule.^74^ For abasic sites or lesioned DNA bases, the incorporation of dA is preferential, but not specific; however, we found that dA is incorporated with high reliability for both M-fC and I-fC. Overall, we showed that during DNA replication, the localized conformation of DNA in the active site is important for recognizing and incorporating the correct or modified base according to the template base. This mechanism is critical for nucleotide recognition by DNA polymerases to maintain replication fidelity.

## 3. DNA structural correlation in short and long ranges

The typical structure of DNA is a right-handed double helix. However, the DNA structure changes in response to variations in its sequence, chemical modification of bases, solvation and interactions with other molecules. For example, adding a methyl group at the 5-carbon site of cytosine (5mC) can lead to distinct DNA structure changes, including narrowed minor grooves, altered conformations of sugar rings, and increased steric hindrance between the major groove and the binding molecules.^75,76^ As another example, the binding of proteins to the major groove of DNA may induce not only an increase in the width of the major groove,^3,4^ but also base flipping^33,45^ or even B- to Z-form DNA transformations.^77^ These DNA conformational changes were shown to be involved in the regulation of many biological processes. MD simulation studies showed that sequence-dependent DNA deformability may be used by the transcription factor to probe the local base sequence.^78,79^ DNA-mediated allostery has been demonstrated to play a crucial role in the control of the DNA–protein interactions.^80–82^ In most cases, the cooperativity in DNA can be explained by a direct read-out mechanism. However, in addition to the direct readout model of allosteric mechanisms, indirect read-out and solvent release mechanisms have also been proposed. In the indirect read-out mechanism, the primary protein distorts the structure of DNA to improve the binding characteristics of the secondary site. The solvent release mechanism assumes that primary binding induces changes in the water or ion distribution, reducing the desolvation cost required for secondary binding.^83^ It was found from a recent simulation study that protein-induced changes in DNA-entropy can also be the origin of cooperativity.^80^ Thus, understanding the DNA structural correlation in short and long ranges and how this correlation can regulate biological processes have attracted significant attention.

**Fig. 4:**
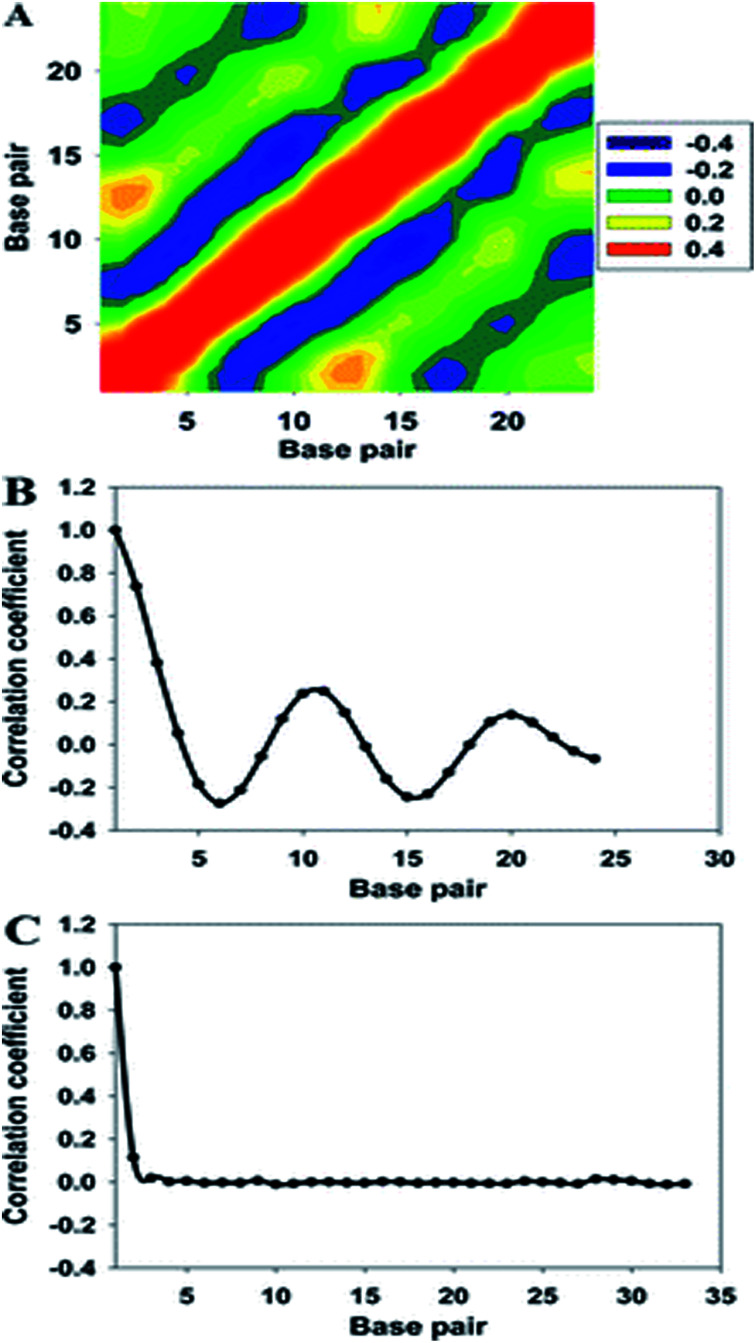
(A) Two-dimensional map of the correlation coefficients of the major groove widths of the 33-bp DNA. High- and low-correlation coefficients are shown in purple and orange, respectively. (B) Averaged correlation coefficients with respect to the major groove width, and (C) correlation function of the diameter of helix. Reproduced with permission from Gu *et al.*, *J. Phys. Chem. B*, 2015, **119**, 13980–13990. Copyright 2015, the American Chemical Society.

To understand the molecular details that affect both the local and relatively long-range structural properties of DNA including allostery, we used a 33-bp DNA segment with the sequence of 5′-GAGATGCTAACCCTGATCGCTGATTCCTTGGAC-3′ as the model system, in which the ratios of the G/C and A/T contents were 51.5% and 48.5%, respectively.^84^ We employed molecular dynamics simulations on the model system and the initial DNA structure was constructed to be in a canonical B form. After energy minimization, heating and equilibrium at 300 K and 1 atm, three independent trajectories were extended to 200 ns for further analysis. Since previous experiments revealed that proteins generally bind to the major grooves of DNA and the binding may induce DNA deformation such as changes in the major groove widths and/or demolition/formation of paired bases, we focused on these two structural deformations and introduced two corresponding structural parameters, namely major groove width and diameter of helix, to measure the magnitude of these two deformations, respectively. Here, the diameter of the helix was defined as the distance between the C3′ atoms of two nucleotides composing the *i*-th base pair. The major groove width was calculated with respect to the *i*-th base pair as the modulus of the vector parallel to the *Z* axis selected from those connecting the C5′ atom of the *i*-th nucleotide to the C5′ and C3′ atoms of the *i* + 3rd to *i* + 9th nucleotides on the complementary strand. In addition, to investigate the correlation of a pair of structural parameters, we introduced the time-averaged correlation coefficients and cross-correlation coefficients as follows:2
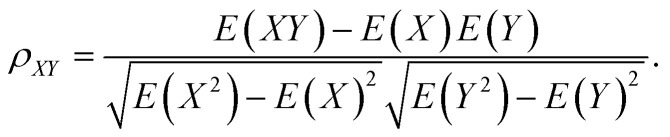
where *ρ*_*XY*_ is the correlation coefficient of interest and *E* is the expectation value operator. For a 33-bp DNA segment, we obtained a 24 × 24 heat map of correlation coefficients when each pair of major groove widths was used (Fig. 4(A)). To clearly show the correlation of the major groove width or diameter of helix between a pair of bases, we averaged the correlation coefficients for an interval *m* between base pair *i* and *i* + *m*. The averaged correlation coefficients of the major groove width – major groove width and those of the diameter of helix – diameter of helix are shown in Fig. 4(B) and (C), respectively. Comparing the variation of the two correlation coefficients, it was easily found that the correlation coefficients of the major groove width oscillate with a periodicity of about 10 base pairs, resembling the result of the binding free energy measured in experiments.^82^ Conversely, this oscillating correlation could not be found for the diameter of the helix. This is a good indication that DNA allostery originates from the change in the major groove width instead of variations of the hydrogen bonding between paired bases. In a recent microsecond-long MD simulation study,^81^ periodicity of the correlation coefficient was not found, and it was proposed that thermally induced long-range vibrations of the same 33-bp DNA double helix are still present at a scale of 10–100 ns, but are already damped out at the microsecond scale. However, a mechanical model of DNA allostery based on constrained minimization of the effective quadratic deformation energy of the DNA captured the ∼10-bp periodicity of protein–DNA allosteric coupling found experimentally.^81^

Furthermore, to find the possible relation between local and long-range structural properties, the correlations between the major groove widths and the fundamental local modes of DNA structure changes were also inspected. As addressed in the Wormlike Chain model,^85–87^ bending, twisting, and stretching are the three most important modes to depict the conformational changes of DNA. However, stretching can be largely restricted by the base stacking in the DNA duplex, which can be speculated to have the least influence on the major groove width. Therefore, we only focused on the average correlation coefficients between the major groove width and twisting or bending. To describe the twisting and bending motions of duplex DNA, we used the base pair step parameter,^88^ twist angle (*ω*), and bending magnitude, which are represented as a vectorial sum of roll (*θ*_R_) and tilt angles (*θ*_T_)3
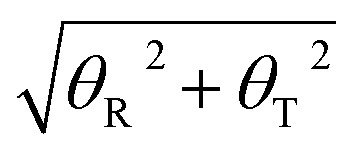


**Fig. 5:**
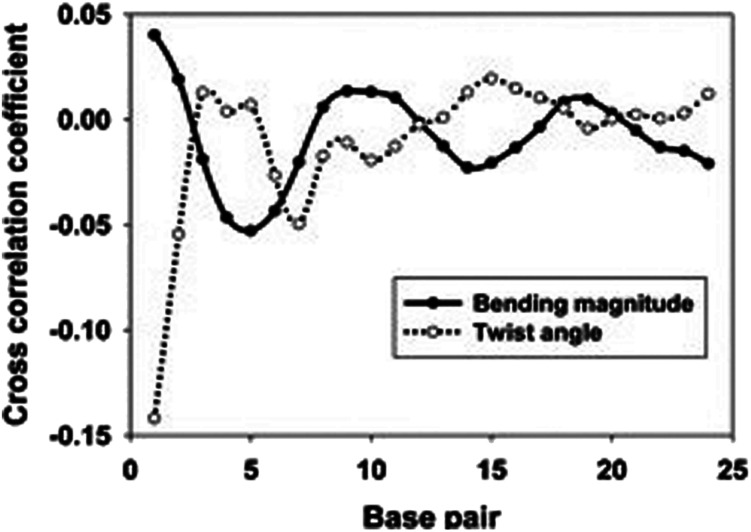
Averaged cross-correlation coefficients between the major groove widths and various structural parameters of the 33-bp sequence. Solid line: cross-correlation coefficients between the major groove and the bending magnitude and dotted line: cross-correlation coefficients between the major groove width and the twist angle. Reproduced with permission from Gu *et al.*, *J. Phys. Chem. B*, 2015, **119**, 13980–13990. Copyright 2015, the American Chemical Society.

As shown in Fig. 5, the cross-correlation coefficients between the major groove width-bending magnitudes showed stronger periodic oscillation than that between the major groove width and twist angle, which indicates the important role of the local bending motion in the DNA allosteric effect.

**Fig. 6:**
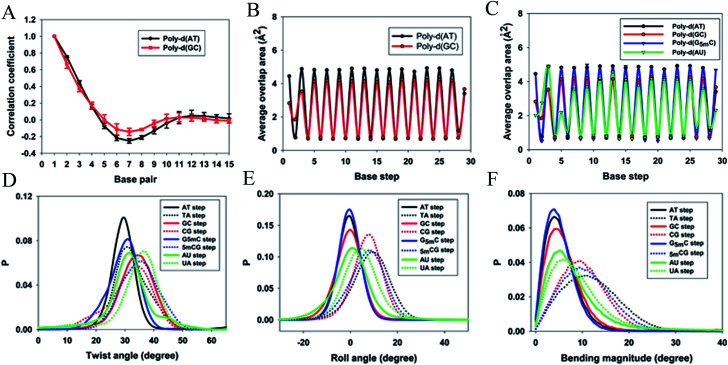
Average (A) correlation coefficients and (B) overlap area for the base steps of poly d(AT) (black line) and poly d(GC) (red line). (C) Average overlap area with respect to each base step of poly d(AT) (black line), poly d(GC) (red line), poly d(G5mC) (blue line), and poly d(AU) (green line). Distribution of (D) twist angles, (E) roll angles, and (F) bending magnitude with respect to the poly d(AT) (black line), poly d(GC) (red line), poly d(G5mC) (blue line), and poly d(AU) (green line). Reproduced with permission from Gu *et al.*, *J. Phys. Chem. B*, 2015, **119**, 13980–13990. Copyright 2015, the American Chemical Society.

When a B-form DNA molecule bends along its *Z* axis, the major groove widths expand and narrow with a periodicity of ∼10 base pairs. The major groove widths on the side with a positive curvature increase, while those on the other side decrease. Protein binding to the major groove could cause local bending of the DNA, which may in turn affect the major groove width. When this bending propagates along the DNA helix, the major grooves away from the first binding site could present widening and narrowing in an alternative and periodic way, affecting the binding of other proteins on different sites of the same DNA. This effect shows a periodicity of ∼10 bp, as described above, and provides a possible explanation for the periodic oscillation of *K*_off_ measured by experiments. Experiments have also shown that the allosteric effect of DNA varies with the DNA sequence. An AT-rich sequence showed a stronger allosteric effect than a GC-rich one. We then examined the sequence dependence of DNA allostery by performing simulations for two model systems, DNAs of poly-d(AT) and poly-d(GC) sequences.^84^ Here, ploy-d(XY) indicates the DNA sequence having alternating dX and dY nucleotides, and d(XY) indicates the XY base pair dinucleotide step of DNA. The calculated averaged correlation coefficients of the major groove widths (Fig. 6(A)) showed a weaker periodic oscillation for poly-d(GC) than for poly-d(AT). Further analysis of the roll and twist angles showed that poly-d(AT) is more prone to bending, while the poly-d(GC) is more prone to twisting. This difference in the preference of local motions induces their difference in DNA allostery. The local structural changes of DNA also provide a structural origin of its sequence dependence. The analyses of the base overlap areas between adjacent base pairs showed that the tendency of twin pair formation is significantly higher for poly-d(AT) than for poly-d(GC) (Fig. 6(B)). However, if methylation is introduced at the C5 position in cytosine, the tendency of twin pair formation can be inverted. We performed MD simulations of DNA with a poly-d(G5mC) sequence and calculated the overlap area between adjacent base pairs.^84^ The overlap area of poly-d(G5mC) exhibits a pattern similar to poly-d(AT) (Fig. 6(C)), namely, upon CpG methylation, poly-d(G5mC) shows a much stronger trend of twin pair formation than poly-d(GC). On the contrary, simulations on poly-d(AU) showed that deoxythymidine demethylation resulted in largely demolished twin pair formation (Fig. 6(C)). In addition, the distributions of twist, roll and bending angles of poly-d(G5mC) and poly-d(AU) showed that the former is easier to twist, whereas the latter bends more easily (Fig. 6(D)–(F)). All the simulation results of the systems with base modifications presented in ref. 84 showed that the base modifications can also alter the conformational transformation of DNA, which further affects DNA allostery. It was found recently that the perturbation generated by a primary protein binding event travels as a wave to distant regions of DNA following a hopping mechanism and the source of allostery is the directionality of time-delayed correlations between the internal degrees of freedom of DNA.^80^

## 4. Structural aspect and gene regulatory function of non-canonical i-motif DNA

The discussion above is mostly related to the canonical DNA structures. However, various non-canonical forms of DNA are also common in cells. The non-canonical DNA structure is associated with the sequence-dependent flexibility of the DNA molecule in solution.^12,14,18^ In many biological processes involving DNA, the DNA double helix is partially unwound into a single-strand sequence. Under certain conditions, repetitive single-strand DNA sequences have the potential to form non-canonical DNA structures such as hairpin, triplex, cruciform, left-handed Z-form, tetraplex (G-quadruplex and i-motif), and A-motif.^12,20,77^ The formation of non-canonical DNA structures has biological consequences considering the fact that repetitive DNA sequences account for more than 50% of the total genomic DNA in the human genome, whereas simple sequence repeats comprise 3% of the total DNA.^89^ Several studies have reported the potential biological relevance of non-canonical DNA structures in mammalian and other genomes considering their gene regulatory function.^12,18,21,90^ Non-Watson–Crick base pairing and alternate hydrogen bonding have significant effects on the folding of non-canonical DNA structures. For instance, non-canonical base pairings between the G–G, C–C, G–G–C and A–A bases in the case of G4, i-motif, triplex and A-motif, respectively, significantly contribute to their structural stabilization and conformational dynamics. Among the different non-canonical DNA structures, the conformational dynamics of G4 and i-motif have received considerable attention due to the recent confirmation of the existence of the G4 and i-motif structures *in vivo* and the presence of the G4- and i-motif-forming sequences in or near the regulatory regions of several genes, especially in the promoter region of oncogene and human telomeric DNA.^18–22,91^ Guanine (G)-rich sequences can form various G4 structures at neutral pH in the presence of cations (Na^+^ and K^+^) in which four guanines are cyclically bound to each other through eight hydrogen bonds according to the Hoogsteen base pairs and form planar quartets. G4 shows significantly distinct parallel and antiparallel structures depending on the type of cation.^12^ It is also well known that G4 shows a high degree of structural polymorphism depending on the nucleotide sequence, the orientation of the strands, the *syn*/*anti* glycosidic conformation of guanines, the loop connectivity, and environmental factors such as cations, molecular crowding and dehydration.^12^

**Fig. 7:**
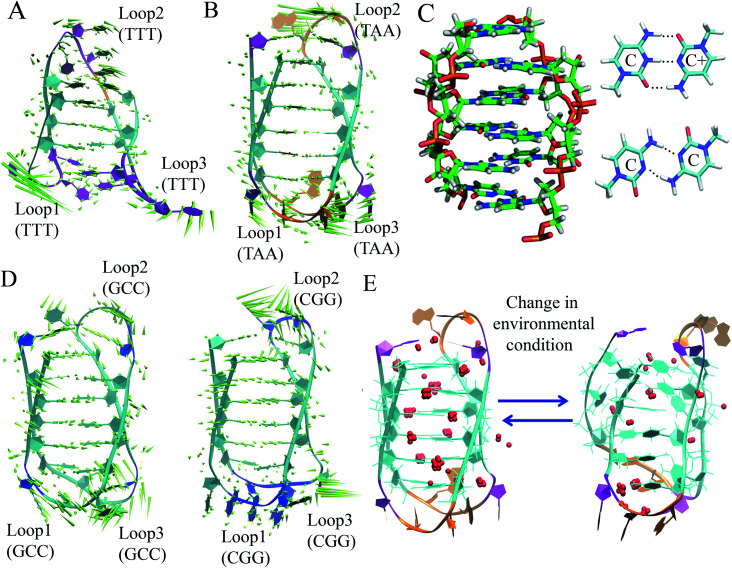
First normal mode of motions in the equilibrated MD trajectory of (A) 3′E and (B) 5′E-form i-motif structure under acidic pH with hemi-protonated cytosines. (C) Base pairing and stacking arrangement within i-motif core. (D) First normal mode of motions in the equilibrated MD trajectory of 5′E_CGG_ (CGG) and 5′E_GCC_ (GCC) form structure having different loop sequences under acidic pH with hemi-protonated cytosines. (E) Alteration of folded conformation and solvent structure around the wide grooves of the 5′E-form i-motif structure in the deprotonated state. Grid water density distribution considering that the water molecules stay in that grid points greater than 0.5 is represented as red spheres.

Although the stability and conformational properties of G4 have been well studied, the *in vivo* formation of the i-motif structure and its biological functions are illusive. The i-motif structure is formed from a cytosine (C)-rich strand. Considering that the *in vitro* characterization of the folded i-motif is closely related to the protonation of the cytosine groups at acidic pH, the formation of the i-motif is believed to be associated with the protonation of the cytosine groups. The latter is beneficial not only for the formation of the hemi-protonated cytosine pairs (C:C^+^)^12,18^ but also cause the cytosine groups to intercalate, and thus four C-rich strands are held together like two parallel duplexes combined in an anti-parallel fashion to form an i-motif.^12,18^ It was found that the stability of the i-motifs is directly linked to the protonation of cytosine, and thus strongly depends on pH. Although the i-motif is mainly formed under slightly acidic conditions, genomic sequences that can form stable i-motif structures at neutral pH have also been reported.^19,22,92^ Several recent studies have shown that reduced temperature, molecular crowding and the presence of cations can influence the formation of the i-motif at neutral pH.^12,18,19,93^ Sugimoto and co-workers reported that triplet repeat sequences, 5′-CGG(CCT)*n*CGG-3' (*n* = 4, 6, 8 and 10), can adopt the i-motif structure at neutral pH by molecular crowding.^93^ Recently, Christ's and Dinger's groups developed an antibody iMab that preferentially binds different well-defined i-motif structures and their study showed that the *in vivo* existence of the i-motif structure is associated with cell cycle progression and transcription.^19^ Dhakal *et al.* observed the coexistence of the partially folded form and i-motif in C-rich human ILPR oligonucleotides using the laser-tweezers technique.^94^ Their study also suggested that the formation of i-motif is decreased by increasing pH, while a small fraction of partially folded structure is pH-independent. MD simulation studies indicated that in the absence of protonated cytosines, hairpin structures are the stable equilibrium conformations at 300 K. The hairpin structure is associated with a free energy barrier of ∼8 kcal mol^−1^ and the fully unfolded strand conformation is energetically less favourable.^95,96^ Depending on the sequence and environmental factors, the i-motifs can fold in different ways to form two different tetramers with different topologies, including one in which the outmost C:C+ pair is at the 3′ end and the other at the 5′ end denoted as 3′E (R-form) and 5′E (S-form), respectively.^18,20^ Potential biologically relevant i-motifs can be formed from natural sequences containing four tracts of cytosines separated by stretches of other bases. One of the most studied cytosine-rich repetitive sequences in the human genome is the telomeric repeat d(CCCTA2). Its complementary G-rich strand forms the G4 structure and is common to various phyla, including vertebrates, fungi, flagellates and slime molds.^18,20^ Based on the nucleotide sequence and loop size, multiple interactions are involved in the folding of the i-motif, together with the interplay of electrostatic interactions with other specific molecular interactions such as hydrogen bonding and base stacking. Thus, a comprehensive study of the interactions that affect the formation, stability and dynamics of i-motifs *in vivo* will help to develop a better understanding of their gene regulatory functions. In this context, our recent MD simulation studies on biologically relevant i-motifs of centromeric and telomeric repeat fragments provided relatively comprehensive atomistic insight into the conformational properties, dynamics, and hydration property of i-motif DNA in acidic and normal pH.^97^ We considered two different topologies, 5′E- and 3′E, and analyzed these two forms under two pH conditions, at neutral pH with the normal unprotonated cytosines and at acidic pH when half of the cytosines are protonated. Using normal mode analysis (NMA) we analyzed the global movements that are collectively encoded by the i-motif fold or topology of native contacts. The normal modes of motions showed that at acidic pH, the overall conformational dynamics of the i-motif is mainly associated with its flexible loop motion (Fig. 7(A) and (B)). The i-motif core is rigid with the distance between consecutive base pairs being 3.1 Å and the right-handed helical twist angle being ∼12–20°, which are both significantly different from canonical B-DNA (Fig. 7(C)). The folded conformation of the i-motif has two narrow grooves and two wide grooves, with the average backbone phosphate–phosphate distances of around 8 Å and 14 Å, respectively. The stability of the i-motif structure is mainly associated with the strong C:C+ pair interaction (−57.6 kcal mol^−1^) within the i-motif core (Fig. 7(C)), as first indicated by Sponer *et al.*^98^ In the deprotonated state, the possible C:C W:W trans base pair (with a base pairing energy of ∼−25 kcal mol^−1^) is found to be unstable within the i-motif core. The structural properties of the i-motif core are mainly governed by the repulsive base stacking interaction. The loop sequence can affect the dynamics and stability of the i-motif by altering its backbone interactions (Fig. 7(D)). At elevated temperatures, the altered motion of the bases in the loop regions is found to affect the folded conformation by altering the backbone interactions along the narrow grooves of i-motif DNA.^97^ We found that i-motif at neutral pH is essentially unstable and unfolds to hairpin structures under thermal fluctuations at the physiological temperature (Fig. 7(E)). Folding and unfolding of the i-motif structure was found to be associated with the alteration in the hydration structure along the wide grooves (Fig. 7(E)).^97^ Similar to the G-quadruplex, the i-motif is also found to show slow folding and unfolding kinetics, which depend strongly on the sequences.^12^ The molecular mechanism of the formation of the i-motif structure *in vivo* at neutral pH is not clearly understood. Considering that cytosine is a major epigenetic target, i-motif forming cytosine-rich sequences may be affected by epigenetic modification. In the recent study by Wright *et al.*, they showed that the stability of the epigenetically modified i-motifs depends on both the type of modification and the cytosine position.^99^ For example, hyper-methylation of a whole cytosine tract was found to disrupt the i-motif that is formed from hTeloC. Moreover, i-motifs that are stable at neutral pH are more likely to be methylated than their acidic counterparts. Molecular level understanding of how these epigenetic modifications impact i-motif structural stability will help us to interpret their biological functions. According to bioinformatics studies, it was found that potential i-motif formation was concentrated in promoters of genes involved in skeletal system development, sequence-specific DNA binding, DNA-templated transcription and positive regulation of transcription by RNA polymerase II.^20,92^ A better understanding of the sequential requirements for the formation of stable i-motifs is necessary to achieve a more accurate mapping of i-motif occurrence along the genome. Therefore, further studies considering cellular environmental factors and different ionic species in solution can provide more insight about the *in vivo* formation of i-motif and its gene regulatory function.

## 5. Molecular basis of DNA condensation and structural organization of chromatin

Besides the structural features of DNA itself, how DNA molecules are assembled and organized in cells is also a fundamental and interesting biological question. In this section, the molecular basis of DNA condensation and structural organization of chromatin, together with the biological functions and regulatory mechanism behind them will be discussed.

### 5.1 Influence of counterion

The structure of chromatin is highly sensitive to the ionic environment, which can play an important role in its compaction under physiological conditions.^29,30,100^ It was found that in the presence of multivalent cations, ion–ion correlations resulted in net DNA–DNA attraction. Another favorable contribution to DNA condensation is the formation of DNA–DNA bridges by oligocations when the ligand is a flexible molecule of sufficient length (*e.g.*, polyamines, N-terminal domains of histones, and protamines).^27,29^ It was found that the self-association of nucleosome arrays in mixed salt solutions is synergistically promoted by Mg^2+^ and monovalent ions, with Na^+^ being slightly more efficient than K^+^. Cations mediating intermolecular interactions function as adhesives between DNA helices and may play a biological role in the crowded DNA media.^101^ Cation binding is sequence dependent and modulates the intrinsic sequence-dependent properties of DNA in terms of the population of conformational states, thus affecting the readout and packaging of DNA.^27,31,32,102^ For example, an A-tract group of sequences (length > 4, AA, TT, or AT base steps with no TA steps) is more likely to adopt the B*-form with a narrow minor groove rich in counterions and G-tracts containing only G and C residues with prominent GG steps are more likely to sample the A-DNA conformation featuring a narrow major groove rich in counterions. All other DNA sequences are considered “generic” and most likely assume the B-form. MD simulations have been used extensively to obtain atomic-level insight into the DNA-counterion interaction. It was found from previous studies that B-DNA can be converted to left-handed Z-DNA under high ionic strength, where the bases are arranged relatively far away from the axis and backbone phosphate groups are placed closer together than B-DNA.^12,103^ The single deep groove of Z-DNA is more hydrated than the grooves of B-DNA. At higher salt concentrations, alternating purine-pyrimidine sequences such as poly-d(GC) are mostly found to adopt the Z-DNA conformation.^12,103^ Under high-salt conditions, the stabilization of the Z-form structure may be due to sequence specific binding and closer clustering of counterions around DNA, which can provide more effective shielding of the mutually repelling backbone phosphate groups. Therefore, sequence specific cation binding has been interpreted as contributing to DNA conformational heterogeneity and linked notably to axial bending and minor groove narrowing. The location of the direct binding sites also depends on the nature of the counterion. In the minor groove of the double helix, the N3 atoms of purines, O2 atoms of pyrimidines, and O4 atoms of deoxyribose are the binding sites for counterions. Conversely, in the major groove of the double helix, the N7 atom of purines is the most important binding site.^31,32^ The microsecond MD trajectories for 39 oligomers containing 136 distinct tetranucleotide base sequences exhibited that the DNA sequence affects the ion populations within the grooves of DNA.^104^ The ion atmosphere around DNA can go beyond the nature of individual base pairs or base pair steps. Within both grooves of DNA, the ion populations at specific base pairs or base pair steps can be strongly influenced by the flanking base sequence.^104^ A MD simulation study also showed that the curvature influences the local environment of DNA, notably *via* increased heterogeneity in the ionic distributions surrounding the double helical DNA.^105^ Various experimental results have shown that divalent cation interactions require dinucleotides containing at least one guanine. Mg^2+^, Ca^2+^, or Mn^2+^ cross-link DNA bases, especially guanines, to phosphates of neighboring helices. Mg^2+^ and Ca^2+^ inter-strand coordination modes have been observed at pure A:T sites and, in some cases, at mixed A:T/G:C sites.^106^ In recent studies, it was found that Mg^2+^ facilitates or enables both the self-assembly of identical double-stranded (ds)DNA molecules and self-assembly of identical nucleosomes *in vitro*.^30,107^ Several aspects of Mg^2+^ functioning as a regulator of chromatin dynamics and chromatin-based biological processes were also revealed.^30^ However, why Mg^2+^ has such a great effect on chromatin condensation compared with monovalent and other divalent cations is poorly understood. More experimental studies and molecular simulations at the atomic level need to be conducted for systems involving chromatins and different types of counterions.

### 5.2 Genomic sequence preference

The dinucleotide distribution in the genomic sequence plays an important role in 3D chromatin structure formation. Based on the densities of dinucleotide sequences, the genome can be divided into large (megabase scale) alternative domains of high and low CpG densities.^24,28^ The CpG-rich/poor regions are associated with compartmentalization, which makes the 3D chromatin structure of higher and lower species distinctly different.^7,24,28,108^ We found that higher species with an uneven dinucleotide distribution tend to have a more segregated chromatin structure than lower ones. The distribution of dinucleotides especially CpG becomes more heterogeneous with evolution. In lower species such as plants and invertebrates, CpG is largely uniformly distributed along the genomes, whereas a mosaic distribution of CpG appears in genomes of higher species such as mammals and birds. Particularly, megabase-scale DNA sequences with a low CpG density and small density fluctuations rarely exist in the genomes of lower species, which only become ubiquitous in the genomes of higher species. The CpG distribution in the genomes of prokaryotes is highly uniform, but the average CpG density varies considerably in different prokaryotes, especially in those living in extreme environments, which leads to distinct sequence properties of prokaryote genomes. For example, the genomes of halobacteria tend to be CpG-rich, which is possibly due to the stability of the CG-rich sequence in a high salt concentration.^7,10^ Therefore, the dinucleotide distribution in the DNA sequence very likely affects the packaging of the genome, and thus it function under different cellular conditions. The sequential difference between different species also shows a correlation with their different responses to the environmental temperature considering that the latter was found to affect the domain segregation in the 3D chromatin structure.^24,28^

**Fig. 8:**
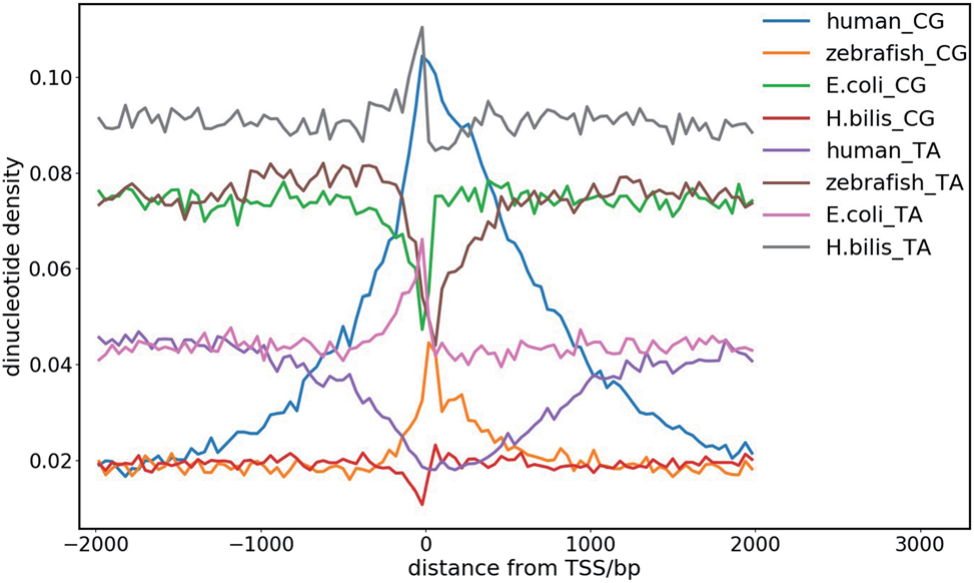
CpG and TpA dinucleotide density distribution at the transcription start sites (TSS) of different species.

The dinucleotide distribution at the transcription start sites (TSS) is also different among species. We found that the DNA sequences in proximity to the TSS of prokaryotes are often CG-poor and TA-rich, while in eukaryotes, they are CG-rich and TA-poor (Fig. 8). The CpG and TpA densities change drastically near the TSS of prokaryotes, which leads to sharp peaks in the CpG and TpA density curves, respectively, whereas the peaks in the dinucleotide density curves near the TSS of eukaryotes are much broader. The AA, AT and TT dinucleotide density variation at and around the TSS was also found to be similar to that of TA. In addition, the dinucleotide distribution near the TSS of genes with different expression patterns also differ. The dinucleotides densities near the TSS of human housekeeping genes change more drastically than that of human tissue-specific genes. Although the absolute values of the dinucleotide density near the TSS vary drastically among species, their change along the genome is conserved among different species. These observations imply that the dinucleotide density gradient may play an important role in the formation of the chromatin structure, transcription initiation and regulation. Consistent with this view, it was found that the AT-rich DNA sequences acquired horizontally in *Escherichia coli* frequently cause constitutive transcription initiation within the coding regions of genes.^109^ It is also known that nucleosomes tend to be excluded from these tracts owing to the rigidity imparted to the DNA by the bifurcating hydrogen bonds between adenosine on one strand and thymine on the other.^110^ Therefore, it is also likely for the dinucleotide density to influence transcription machinery assembly at promoters through nucleosome positioning.

A necessary step towards understanding the formation of the chromatin structure and DNA condensation at the molecular level is the characterization of the physical properties of DNA, including the effects of the nucleotide sequence, chemical modifications and environmental factors, such as temperature, pH and ionic strength. A recent study proposed that the differential affinity between the DNA regions of varying sequence patterns may drive the phase separation of chromatin into chromosomal subdomains.^27^ MD studies showed that the strength of DNA condensation depends not only on the nucleotide composition but also on the local DNA sequence.^27^ AT-rich DNA duplexes associate more strongly than GC-rich duplexes, regardless of the sequence homology. Methylation of cytosines was found to induce attraction between GC-rich DNA as strong as that between AT-rich DNA.^27^ Recent genome-wide chromosome organization studies showed that remote contact frequencies are higher for AT-rich and methylated DNA, which suggest that direct DNA–DNA interactions may play a role in the chromosome organization and gene regulation. It was proposed that the local nucleotide content may potentially play a role in the structural organization of eukaryotic chromosomes.^24,27^ The genomic sequence preference among different species and structural organization of chromatin were also found to correlate with body temperature control.^24,28^ Therefore, future atomistic studies in this context considering different environmental factors and double-stranded DNA fragments having different dinucleotide sequences and counterions will help to understand the sequence preference in the phase separation mechanism, chromatin folding, dynamics and organization, which are associated with transcription, gene regulation and cell function.

## 6. Summary and outlook

The conformational properties and dynamics of canonical and non-canonical DNA in physiological conditions are intimately related with their biological functions. Base sequences and environmental factors, such as chemical modification, temperature, ionic strength and pH affect the formation and stability of the three-dimensional structure of DNA. The structural variability and flexibility of DNA are mainly governed by its base sequence, and base pairing and stacking interactions primarily help to maintain the different folded conformations of DNA. The sequence-dependent structure and dynamics of DNA were revealed from extensive MD simulation studies *via* a divide and conquer approach by the Ascona B-DNA Consortium (ABC) considering all the possible tetra-nucleotide repeats of DNA.^111–113^ The results suggested that the sequence dependence of dinucleotides is significant and that the intrinsic flexibility of the YR steps is higher than that of the RR (YY) and RY steps (R represents purine and Y represent pyrimidine). Moreover, the dinucleotide conformation can be strongly affected by the flanking bases. It was found from microsecond-long simulations that many sequences occupy more than one conformational sub-state at physiological temperature, having different helical parameters, and the base sequence modifies DNA fluctuations in a selective manner, often affecting only one or a small subset of helical parameters. The hydration property and presence of counterions can also influence the sequence-dependent conformational preference of DNA.^32,102,104,105,114,115^

The chemical composition of the four base types of DNA may seem surprisingly simple, but the storage, organization and usage of genetic information are highly complex and many hierarchical orders exist, ranging from single bases to chromosomes. The physical properties of DNA at these different scales are all likely to contribute to its biological function and regulation. Information at the single base level, such as GC content, at the dinucleotide level, such as the CpG base step, at the oligomer level, such as TATA box and longer sequences ranging from poly A tract to CpG island (CGI), and even higher order sequence information such as the isochores or CGI forests and prairies^24,28,108^ have all been shown to correlate with the realization of biological functions. The higher order structure formation of chromatin has been found to be dependent on the DNA sequence, and in many cases, simple sequence properties such as nucleotide and dinucleotide densities.^24,28^ Large-scale sequence segregation in the 3D space resembling phase separation affects multiple biological processes such as transcription and replication.

The motions and structures involved in the related biological processes are also multi-scale, ranging from single base flipping to local DNA deformation, transcription factor (TF) binding, G-quadruplex and i-motif formation, nucleosome formation, DNA looping, self-interacting genomic region or topologically associating domain (TAD) establishment, compartmentalization, and even chromosome territory formation. The sequence-dependent physical properties of DNA play vital roles in all these events, and thus it is interesting to examine how simple information such as nucleotide and dinucleotide density affects DNA and the formation of the chromatin structure in these different hierarchical orders. It is reasonable to speculate that the more general structure features such as open chromatin and compartment formation depend heavily on the average properties of the DNA sequence such as CpG density, whereas the realization of specific functions is dependent strongly on longer sequence motifs.

Base pair opening or base flipping is a crucial local distortion associated with DNA repair and epigenetic imprinting, which is mediated by the specific DNA processing enzymes. The base of a single mismatched base pair in a double-stranded DNA can spontaneously flip out of the DNA duplex, which lays the foundation to fully understand exactly how the repair proteins search and locate the target mismatched base among a vast excess of matched DNA bases. Employing SITS molecular simulations, we gained insight into the role of the conformational fluctuations of protein in the enzymatic base flipping mechanism, including the energetic and flipping pathways of the bases in the mismatched base pairs. The lifetime of the tested mismatched bases stayed at an extra-helical position far away from their intra-helical state, suggesting that the mismatched bases spend sufficient time outside the DNA duplex. Our results suggested that the capture of a flipped-out base by repair proteins is possible. The real damage-searching mechanism may be multifaceted, involving a combination of capturing of spontaneously flipped out base and probing of weakened base pairing to maximize damage searching efficiency.^37,38,40^ The base flipping mechanism and kinetics may vary depending on the DNA sequence, classes of DNA modifying or repair enzymes and nature of the mismatch or damaged bases. Thus, future studies in this context will help in understanding the underlying base-flipping mechanism and active or passive involvement of the protein in enzyme-catalyzed DNA processing mechanism.

On a larger scale, molecular dynamics simulations of DNA oligomers led us to propose that the long-range DNA allostery is linked to its bending flexibility. The bending flexibility of DNA is sequence dependent and is affected by the existence of weak points of base stacking along the helix. One of the most significant effects of chemical modification of bases is the alteration of the base stacking mode to affect the long-range conformational changes. The analyses of the model systems also showed that DNA acts as more than a simple docking site for proteins, but participates actively in gene regulation through shape changing in response to chemical modifications. The deformation of DNA is associated with its sequence-dependent flexibility, but exact mechanism by which DNA deforms its geometry upon protein binding is not understood very well, where MD simulation results support either the induced fit or the conformational selection paradigms.^3,4,42,116^ Transcription factors are the core element of the gene regulatory network and it was found that the DNA deformability may be used by the transcription factors to probe the local base sequence.^2,3,117^ Systematic studies on the sequence and chemical modification dependence of the structural properties of DNA, including the allosteric effects and their influence on TF binding are highly desirable.

It is of great interest to understand the specific structure formation on scales of tens to hundreds of base pairs given that this length is pertinent to TF binding and open chromatin formation. As one example, cytosine- or guanine-rich repetitive DNA sequences that can form non-canonical structures are found in telomeres and promoter regions of several oncogenes, where alterations in the non-canonical structures are argued to play important roles in the progression of cancer and other diseases. The conformational dynamics and folding/unfolding of the G4/i-motif structure have an important biological consequence considering their gene regulatory function. It was found that the presence of G4s/i-motifs enhances myoD-dependent gene expression, and transcription of the c-myb gene is suppressed by G4/i-motif formation.^12,18^ In some cases, it was found that i-motif may act as a more effective inhibitor of DNA replication than G4 or hairpin structures, even though they have similar thermodynamic stabilities.^12^ The conformational dynamics of non-canonical DNA reported to date have shown diverse results. Most of the recent studies indicate that the folding and unfolding dynamics of non-canonical DNA prefer multi-pathways with detectable intermediates rather than a simple two-state process. Detailed knowledge about the conformational dynamics and folding/unfolding mechanism of the non-canonical DNA structure is required to understand its gene regulatory function. However, although several studies have been conducted to date, the connection between the physical properties of DNA and gene regulatory mechanisms is still an unknown part of the genetic code.

The long range correlations of the DNA primary sequences with their 3D structures indicate base sequence can affect the folding and formation of the higher order structure of DNA. From recent experimental observations, it was found that the 3D structure of chromatin is associated with the structural domains at different scales (*e.g.*, loops, TADs and compartments), which is important for gene regulation. Interestingly, the distinction between the compartments strongly correlates with the local variation of TA richness. The gene-poor domains that have a lower CG content on average are assembled near the nuclear membrane, while the gene-rich domains with a higher CG content are grouped near the inner space of the nuclei. In this context, our observation on the spatial segregation of sequence-specific CG-rich and poor domains indicates a phase separation mechanism in the formation of the chromatin structure and remodeling.^24,28^ Similar to the importance of the amino acid sequence in the protein structure and function, the nucleotide sequence can be the driving force for the phase segregation and structural organization of chromatin, although the latter involves much more complex interactions, including DNA/protein interactions. The dinucleotide density distribution at and near the TSS site of the genome can be associated with the chromatin folding and gene regulation. The sequence-based chromatin segregation and mixing mechanisms together shape the chromatin organization in different biological processes and are important for biological functions. The structural organization of chromatin and its correlation with gene regulation and development are evident from the genomic sequence studies^24,28^ and recent experimental observations;^23,25,118^ however, microscopic basis of the DNA sequence-mediated phase separation mechanism and its role in hierarchical chromatin structural formation and organization needs to be further revealed. It would also be intriguing to further investigate the physical properties of DNA considering the important biological consequences of temperature, ionic strength and protein–DNA interaction, which can regulate the folding, dynamics and organization of chromatin in the cell.

The last few years have witnessed tremendous progress in MD simulation applied to nucleic acids due to the improvement of simulation algorithms, solvent models and force fields.^78,111,119^ Simulation of DNA has evolved under the impetus afforded by the increase in computing power, making longer simulations possible. The reliability of force fields for describing DNA flexibility is a critical aspect due to the scarcity of experimental data. However, there are certain limitations of currently available and reliable force fields for nucleic acids.^78,119^ In microsecond timescale simulation, the robustness of currently available nucleic acid force fields is still an unsolved question. Certain versions of nucleic acid force fields fail to reproduce proper backbone conformations, structural transitions, and special loop structures, and no guarantee exists that they can capture some non-helical conformations of DNA. Thus, a critical evaluation of the current nucleic acid force fields is still required. Choice of the nucleic acid force fields has been found to influence the simulation results.^119,120^ It is desirable for similar structural properties and dynamics to be observed with multiple force fields, each derived independently and with a different viewpoints to further support the validity of the MD simulation results. The MD picture of the ionic atmosphere around DNA remains controversial due to the reliability of the empirical force–field parameters, and, in most cases, the absence of a polarizability term. Due to the issue of lack of convergence in nanosecond-scale simulations, the slow diffusion of ions in water may cause problems.^104^ Furthermore, the simulation of divalent ions, such as Mg^2+^, Mn^2+^, and Zn^2+^, which are important in certain DNA structures, is unfortunately very difficult using pairwise potentials.^78,121^ Thus, further improvements in force field by recalibration of non-bonded functionals including explicit polarization are necessary for the simulation of divalent ions. Conversely, the development of a polarizable force field for nucleic acids is also an active field of research.^122^ Simulation study of very long DNA fragments in longer timescales further demands the development of coarse-grained force fields, which is at the expense of a loss of resolution to allow the fast calculation of the mechanical properties and dynamics of DNA. Further advances in this field will probably also arise from the improvement of computer resources, which will allow the atomistic detail study of the nucleic acid structure, dynamics and interactions in a longer timescale, from the development and implementation of enhanced sampling methods.

As discussed in this perspective, the deformation and dynamics of DNA in different length and timescales have vital implications in fundamental biological processes and an in-depth molecular level understanding of their role in various DNA functions is still necessary. Besides its sequence-dependent flexibility, the groove geometry, hydration and related dynamics of DNA are vital for many biological processes, which need to be elucidated further. The local deformation of DNA and complex dynamics of hydrating water and ions in and around DNA are found to be generally dependent on the DNA sequence. However, the sequence specific cation binding, hydration properties and ion-specific conformational properties of DNA, which are still largely overlooked, may play an important role in DNA recognition and binding. The combined effects of a physiologically relevant mixed ionic environment of K^+^, Mg^2+^ and Na^+^, which are the main cations of the cell cytoplasm, have not been systematically investigated. There are strong correlations between asymmetry in cation localization, DNA groove geometry, and DNA curvature.^104,105^ However, the role of the counterion distribution remains unknown or controversial. The thermodynamic affinity of DNA for counterions can be substantially changed by interactions with numerous DNA-binding proteins, by effects of DNA bending in the nucleosome, and by a number of other factors, which have not been systematically investigated. Thus, we believe that combined with experimental efforts, multi-scale modelling, enhanced sampling and large-scale MD simulation studies will help to provide atomistic insight into the complex biological functions involving DNA in the years to come.

## Conflicts of interest

There are no conflicts to declare.
